# Emerging Insights Into Necroptosis: Implications for Renal Health and Diseases

**DOI:** 10.7759/cureus.43609

**Published:** 2023-08-16

**Authors:** Anannya Gupta, Swarupa Chakole, Suyash Agrawal, Harshal Khekade, Roshan Prasad, Tejaswee Lohakare, Mayur Wanjari

**Affiliations:** 1 Internal Medicine, Jawaharlal Nehru Medical College, Datta Meghe Institute of Higher Education and Research, Wardha, IND; 2 Community Medicine, Jawaharlal Nehru Medical College, Datta Meghe Institute of Higher Education and Research, Wardha, IND; 3 Medicine, Jawaharlal Nehru Medical College, Datta Meghe Institute of Higher Education and Research, Wardha, IND; 4 Child Health Nursing, Smt. Radhikabai Meghe Memorial College of Nursing, Wardha, IND; 5 Research and Development, Jawaharlal Nehru Medical College, Datta Meghe Institute of Higher Education and Research, Wardha, IND

**Keywords:** therapeutic targeting, inflammation, molecular signaling pathways, cell death, renal disease, renal health, necroptosis

## Abstract

Necroptosis is a regulated form of cell death that has gained increasing attention in recent years. It plays a significant role in various physiological and pathological processes, including renal health and disease. This review article provides an overview of necroptosis as a regulated cell death pathway and explores its implications in renal physiology and renal diseases. The molecular signaling pathways involved in necroptosis, including the key players such as receptor-interacting protein kinases (RIPKs) and mixed lineage kinase domain-like protein (MLKL), are discussed in detail. The crosstalk between necroptosis and other cell death pathways, particularly apoptosis, is explored to understand the interplay between these processes in renal cells. In normal physiological conditions, necroptosis has been found to play a crucial role in renal development and tissue homeostasis. However, dysregulated necroptosis can contribute to tissue damage, inflammation, and fibrosis in renal diseases. The review highlights the involvement of necroptosis in acute kidney injury, chronic kidney disease, and renal transplant rejection, elucidating the underlying pathophysiological mechanisms and consequences. The therapeutic targeting of necroptosis in renal diseases is an emerging area of interest. Current and emerging strategies to modulate necroptosis, including the inhibition of key mediators and regulators, are discussed here. Additionally, the potential therapeutic targets and inhibitors of necroptosis, along with preclinical and clinical studies exploring their efficacy, are reviewed.

## Introduction and background

Cell death, a fundamental biological phenomenon, is pivotal in diverse physiological and pathological contexts. In addition to the extensively studied apoptosis, which represents a classical form of programmed cell death, recent advances in research have unveiled insights into another intricately regulated process known as necroptosis. Necroptosis is a complex interplay between necrosis and apoptosis, demonstrating unique regulatory mechanisms. A specific molecular cascade that centers around the activation of receptor-interacting protein kinases (RIPKs) is central to the orchestration of necroptosis. These kinases, including notable members such as RIPK1 and RIPK3, are crucial in driving the necroptotic pathway. By better understanding the intricate triggers and activators of RIPKs, we can enhance our appreciation of the nuances within this process. Hence, this article delves into the intricate web of molecular events that lead to the activation of RIPKs and their subsequent engagement with the executioner molecule, mixed lineage kinase domain-like protein (MLKL) [1-5]. This insight deepens our comprehension of necroptosis and sheds light on potential therapeutic avenues for manipulating this pathway in various disease contexts.

The importance of necroptosis in various organ systems has been increasingly recognized, including its implications for renal health and disease. The kidney is vital for maintaining fluid and electrolyte balance, regulating blood pressure, and eliminating waste. Disruption of renal homeostasis can lead to the development of renal diseases, such as acute kidney injury (AKI) and chronic kidney disease (CKD). Recent studies have uncovered the involvement of necroptosis in renal pathophysiology, highlighting its potential as a therapeutic target [6-8].

This review article aims to provide an in-depth overview of necroptosis as a regulated form of cell death and its significance in renal health and disease. By exploring the molecular mechanisms of necroptosis, its role in renal physiology, and its contribution to various renal diseases, we aim to enhance our understanding of the complex interplay between necroptosis and renal function. Furthermore, we will discuss the potential therapeutic strategies that target necroptosis for treating renal diseases.

## Review

Mechanisms of necroptosis

Molecular Signalling Pathways Involved in Necroptosis

The process of necroptosis is set into motion by specific cell surface receptors, notably tumor necrosis factor receptor 1 (TNFR1), Toll-like receptors (TLRs), and interferon receptors. Activation of these receptors initiates a sequence of molecular events that assemble a necrosome complex. Within this necrosome, key players like receptor-interacting protein kinases take center stage, orchestrating the initiation and execution of necroptosis [9-11].

Upon receptor activation, RIPK1 is recruited to the receptor complex, where it undergoes crucial phosphorylation events and provides a scaffold for recruiting other pivotal signaling molecules. Subsequently, RIPK3 joins the necrosome, facilitated by its interaction with phosphorylated RIPK1. This interaction prompts the activation and self-phosphorylation of RIPK3, triggering the formation of either a RIPK1-RIPK3 heterodimer or RIPK3 oligomers [12,13].

Key Players: Receptor-Interacting Protein Kinases, Mixed Lineage Kinase Domain-Like Protein, and Other Components

RIPK1 and RIPK3 are critical regulators of necroptosis. RIPK1 not only participates in the necrosome formation but also regulates the balance between necroptosis and apoptosis. Depending on the cellular context and availability of other signaling molecules, RIPK1 can form complexes with either RIPK3 or Fas-associated death domain protein (FADD), leading to the activation of necroptosis or apoptosis, respectively [11,12,14]. The phosphorylation of RIPK3 by RIPK1 promotes the recruitment and activation of MLKL. The activated MLKL undergoes conformational changes and translocates to the plasma membrane, disrupting membrane integrity and leading to cellular rupture and necroptotic cell death [15].

In addition to RIPK1, RIPK3, and MLKL, other components, such as cylindromatosis (CYLD), caspases, and phosphoglycerate mutase family member 5 (PGAM5), also contribute to the regulation of necroptosis. CYLD acts as a negative regulator of necroptosis by deubiquitinating RIPK1 and RIPK3, thereby preventing their activation. Conversely, caspases can cleave and inactivate RIPK1 or RIPK3, shifting the balance towards apoptosis. PGAM5, a mitochondrial protein, has been implicated in necroptosis by modulating mitochondrial function and reactive oxygen species (ROS) production [16,17].

Crosstalk Between Necroptosis and Other Cell Death Pathways

Necroptosis and apoptosis are interconnected and share several signaling components. The balance between these two pathways is regulated by the availability and activity of key molecules such as RIPK1, FADD, and caspases [18]. Under conditions where apoptotic signaling is inhibited or caspases are inactive, necroptosis can be engaged as an alternative cell death pathway. In some scenarios, necroptosis can also be triggered due to failed apoptosis, referred to as "necroptosis backup." Additionally, the engagement of necroptosis can lead to the release of pro-inflammatory molecules, promoting inflammation and further influencing cell death pathways [19].

The crosstalk between necroptosis and apoptosis is complex and context-dependent. Various factors, including cellular context, molecular availability, and inflammatory signals, determine the outcome and preference for one pathway over the other [20]. Understanding the molecular signaling pathways and the interplay between necroptosis and other cell death pathways, particularly apoptosis, is essential for unraveling the complex mechanisms underlying cell fate decisions in renal health and disease. Such insights will help identify novel therapeutic targets and develop strategies to modulate necroptosis for the treatment of renal disorders.

Necroptosis and renal physiology

Normal Physiological Roles of Necroptosis in Renal Development and Tissue Homeostasis

Necroptosis is associated with pathological conditions and is important in normal renal physiology. During renal development, necroptosis contributes to tissue remodeling and sculpting, ensuring the proper formation of the kidney architecture. Selectively eliminating specific cell populations through necroptosis is crucial for precisely shaping renal structures, such as nephrons and collecting ducts [21]. In adult kidneys, necroptosis serves as a mechanism for maintaining tissue homeostasis. It helps to eliminate damaged or senescent cells, preventing the accumulation of dysfunctional cells and maintaining renal function. Necroptosis also increases renal cell turnover, ensuring renal epithelium renewal and overall tissue integrity [22].

Autophagic cell death (type II) involves a process known as autophagy, where cells undergo a controlled self-digestion of their components in response to various stresses. This mechanism is distinct from apoptosis and necrosis, but interactions and crosstalk between these pathways have been observed. Autophagy plays a significant role in maintaining cellular homeostasis and promoting survival by recycling cellular components and providing energy during nutrient scarcity [18-21].

There is emerging evidence of complex interactions in the context of necroptosis and autophagy. Some studies suggest that autophagy can promote necroptosis by providing the necessary cellular components or conditions for its execution. Conversely, in certain scenarios, autophagy might act as a protective mechanism against necroptosis by clearing damaged organelles or proteins [22-24]. In renal health, the interplay between necroptosis and autophagy gains significance. Proper autophagic processes are crucial for maintaining the health and function of renal cells, as they help clear cellular debris and prevent the accumulation of damaged components. Dysregulation of autophagy or an improper interaction between necroptosis and autophagy could contribute to renal dysfunction or disease development.

Regulation of Necroptosis in Renal Cell Types

Different renal cell types exhibit distinct susceptibilities and regulations of necroptosis. Tubular epithelial cells, the primary cell type in the renal tubules, are particularly sensitive to necroptosis. Various stimuli, such as ischemia-reperfusion injury, nephrotoxic agents, and oxidative stress, can trigger necroptosis in tubular epithelial cells, leading to renal dysfunction [23]. Glomerular cells, including mesangial and podocytes, are susceptible to necroptotic cell death. Dysregulated necroptosis in glomerular cells has been implicated in the development and progression of glomerular diseases, such as glomerulonephritis and diabetic nephropathy [24].

In addition to tubular epithelial and glomerular cells, interstitial cells, including fibroblasts and immune cells, can undergo necroptosis in response to inflammatory insults or tissue damage. The dysregulation of necroptosis in interstitial cells can contribute to the amplification of inflammation and the progression of renal fibrosis [25].

Interplay Between Necroptosis and Inflammation in the Kidney

Necroptosis and inflammation are tightly interconnected processes in the kidney. Necroptotic cell death can trigger the release of intracellular contents, known as damage-associated molecular patterns (DAMPs), which function as danger signals and activate innate immune responses. DAMPs, including high-mobility group box 1 (HMGB1), mitochondrial DNA, and various cytokines, recruit immune cells, such as macrophages and dendritic cells, and promote the production of pro-inflammatory mediators [26]. Conversely, inflammation can influence the susceptibility and regulation of necroptosis in the kidney. Inflammatory signals, such as cytokines and chemokines, can enhance the activation of necroptotic pathways and sensitize renal cells to necroptotic stimuli. Inflammatory cells, such as macrophages, can release factors that promote necroptosis in neighboring renal cells [27].

The interplay between necroptosis and inflammation in the kidney forms a positive feedback loop, leading to the perpetuation of renal injury and the progression of renal diseases. Understanding the intricate relationship between necroptosis and inflammation is crucial for developing strategies to modulate these processes and mitigate renal damage in various pathological conditions [28]. The insights gained from studying the normal physiological roles of necroptosis in renal development and tissue homeostasis and the regulation of and interplay between necroptosis and inflammation in different renal cell types provide a foundation for elucidating the involvement of necroptosis in renal diseases. This knowledge can pave the way for developing targeted therapeutic interventions to preserve renal health and ameliorate renal disorders.

Necroptosis in renal diseases

AKI and Necroptosis: Pathophysiological Mechanisms and Consequences

Acute kidney injury is characterized by a rapid decline in renal function, often resulting from ischemia-reperfusion injury, sepsis, or nephrotoxic insults. Necroptosis has emerged as a significant contributor to AKI pathogenesis. Ischemia-reperfusion injury, a common cause of AKI, triggers necroptotic cell death in renal tubular epithelial cells. Activating necroptotic signaling pathways, including RIPK1, RIPK3, and MLKL, leads to cellular demise and the release of pro-inflammatory mediators, DAMPs, and cytokines [29].

Necroptosis-mediated tubular cell death in AKI contributes to direct renal damage and initiates a cascade of inflammatory responses, leading to the recruitment and activation of immune cells. The release of DAMPs and pro-inflammatory cytokines activates innate immune receptors, triggering an inflammatory response that exacerbates renal injury. Furthermore, necroptosis can impair tubular regeneration and delay the recovery of renal function following AKI [30].

Chronic Kidney Disease and Necroptosis: Involvement in Progression and Fibrosis

CKD is characterized by progressive renal function loss and interstitial fibrosis development. Necroptosis has been implicated in the pathogenesis and progression of CKD. Persistent renal insults, such as inflammation, oxidative stress, and metabolic abnormalities, can trigger necroptosis in renal cells, particularly tubular epithelial cells [31].

Necroptosis-mediated tubular cell death in CKD contributes to the release of pro-inflammatory mediators and DAMPs, leading to the recruitment of immune cells and the perpetuation of renal inflammation. The persistent activation of necroptosis in tubular cells also triggers fibrotic responses, promoting the activation of fibroblasts and the deposition of extracellular matrix proteins [32].

The interplay between necroptosis, inflammation, and fibrosis in CKD forms a vicious cycle, resulting in the progressive loss of renal function and the development of end-stage renal disease. Targeting necroptosis pathways holds promise for preventing or attenuating renal fibrosis and halting the progression of CKD.

Renal Transplant Rejection and Necroptosis: Insights Into the Immune Response

Renal transplant rejection remains a significant challenge in solid organ transplantation. Emerging evidence suggests that necroptosis involves the immune response and allograft rejection. Following transplantation, various factors can trigger necroptosis in the transplanted kidney, including ischemia-reperfusion injury, immune-mediated damage, and inflammatory signals [33]. Necroptosis in renal transplant rejection influences the activation and recruitment of immune cells, including T cells and macrophages, and the release of pro-inflammatory cytokines. Necroptosis-mediated release of DAMPs and cytokines promotes immune cell infiltration and alloreactivity, contributing to the destruction of the transplanted kidney [34].

Understanding the role of necroptosis in renal transplant rejection provides insights into the immune response and potential targets for therapeutic intervention. Strategies to modulate necroptosis pathways could help mitigate allograft rejection and improve long-term outcomes in renal transplantation [35]. The involvement of necroptosis in various renal diseases, including AKI, CKD, and renal transplant rejection, highlights its significance as a potential therapeutic target. By elucidating the pathophysiological mechanisms and consequences of necroptosis in these conditions, researchers can develop novel therapeutic strategies to prevent or mitigate renal damage, promote tissue repair, and improve clinical outcomes [36].

Therapeutic targeting of necroptosis in renal diseases

Current and Emerging Strategies to Modulate Necroptosis

Modulating necroptosis holds promise as a therapeutic strategy for renal diseases. Several approaches have been explored to target necroptotic pathways. One approach is to inhibit the key mediators of necroptosis, such as RIPKs and MLKL. Small-molecule inhibitors and genetic approaches have been developed to target these proteins and interfere with their activation or function [37].

Another strategy involves targeting upstream regulators or signaling molecules involved in necroptosis. For example, inhibitors of TNF signaling or TLRs can be used to prevent the initiation of necroptotic signaling cascades. Modulating the expression or activity of negative regulators of necroptosis, such as cylindromatosis, can also be explored as a therapeutic approach [38]. Furthermore, targeting the inflammatory response associated with necroptosis can be beneficial. Inhibiting pro-inflammatory cytokines, such as interleukin-1β (IL-1β) or TNF-α, can attenuate the amplification of necroptosis and inflammation in renal diseases.

Potential Therapeutic Targets and Inhibitors of Necroptosis

Several potential therapeutic targets and inhibitors of necroptosis have been identified. Small-molecule inhibitors targeting RIPK1, such as necrostatins and their derivatives, have shown efficacy in experimental models of renal diseases. The inhibition of RIPK3 using small-molecule inhibitors, such as GSK'872 and dabrafenib, has also demonstrated promising results [39].

MLKL, the executioner protein in necroptosis, has been targeted using inhibitors such as necrosulfonamide (NSA) and GW806742X. Additionally, inhibitors targeting upstream regulators of necroptosis, including TLR inhibitors, TNF inhibitors, and CYLD activators, are being investigated as potential therapeutic agents [40].

Preclinical and Clinical Studies Exploring the Efficacy of Necroptosis Modulation in Renal Diseases

Preclinical studies using animal models of renal diseases have provided evidence supporting the therapeutic potential of necroptosis modulation. These studies have shown that the inhibition of necroptosis can attenuate renal injury, reduce inflammation, and improve renal function in AKI and CKD models. Moreover, in renal transplant models, targeting necroptosis has been shown to alleviate allograft rejection and improve graft survival [41]. While necroptosis modulation in renal diseases is still in its early stages, there is growing interest in exploring the clinical translation of these findings. Clinical studies evaluating the efficacy and safety of necroptosis-targeted therapies in renal diseases are needed to validate the preclinical findings. These studies will provide valuable insights into the feasibility and potential benefits of necroptosis modulation in the clinical management of renal diseases [42].

Molecular Basis of Therapeutic Interventions Targeting Necroptosis in Renal Diseases

Inhibition of necroptosis pathway components: One approach involves targeting key components of the necroptosis pathway, such as RIPK1 and RIPK3. Small-molecule inhibitors or molecular tools that block the kinase activity of RIPK1 or the interaction between RIPK1 and RIPK3 have shown promise in preclinical studies. These inhibitors can prevent necrosome formation and halt the downstream events that lead to necroptosis [9-11].

Utilizing kinase inhibitors: Small-molecule inhibitors that target upstream kinases involved in necroptosis initiation, such as MLKL, have also been explored. By inhibiting MLKL phosphorylation or its oligomerization, these compounds can disrupt the downstream signaling events that trigger necroptosis [12].

Modulation of RIPK1 functions: Apart from its role in necroptosis, RIPK1 is a multifunctional protein involved in various cellular pathways, including apoptosis and inflammation. Selective targeting of RIPK1 functions has emerged as a therapeutic strategy. By blocking RIPK1's pro-necroptotic activity while preserving its anti-apoptotic and pro-survival roles, it is possible to influence cell fate decisions in favor of survival [11].

Autophagy modulation: As discussed earlier, the interplay between necroptosis and autophagy is intricate. Manipulating autophagy through pharmacological agents or genetic interventions could potentially influence cells' susceptibility to necroptosis. Enhancing autophagy may promote the clearance of damaged cellular components, reducing the triggers for necroptosis [14].

Combination therapies: Given the complexity of necroptosis and its interactions with other cell death pathways, combination therapies could hold promise. Combining necroptosis inhibitors with agents targeting related pathways, such as apoptosis or inflammation, might yield synergistic effects and improve therapeutic outcomes [15].

Specific targeting in renal diseases: Tailoring these interventions to the unique molecular and cellular context of different renal diseases is crucial. Understanding the specific triggers and molecular players involved in necroptosis in conditions like acute kidney injury, chronic kidney disease, or glomerulopathies is necessary to design effective therapies [16].

Future perspectives and challenges

Areas Requiring Further Research and Understanding

Elucidating the precise molecular mechanisms underlying necroptosis in different renal cell types: Further investigation is needed to unravel the cell type-specific signaling pathways and molecular interactions that govern necroptosis in various renal cells. This understanding will enable the development of more targeted therapeutic approaches.

Identifying additional regulators and effectors of necroptosis: Although the core components of the necroptotic pathway have been identified, additional regulators and effectors may not be discovered. Uncovering these molecules will enhance our understanding of the complexity of necroptosis and provide new targets for intervention [43].

Exploring the role of necroptosis in specific renal diseases: While the involvement of necroptosis has been demonstrated in AKI, CKD, and renal transplant rejection, its role in other renal diseases, such as glomerular diseases and inherited renal disorders, remains to be fully elucidated. Further studies are needed to determine the extent of necroptosis involvement in these conditions.

Limitations of Current Studies and Potential Solutions

Lack of specific inhibitors and tools: While several small-molecule inhibitors targeting necroptosis components have been developed, their specificity and off-target effects must be carefully evaluated. Developing more specific inhibitors and tools will enable researchers to dissect the role of necroptosis more accurately.

Limited clinical data: Clinical studies evaluating the efficacy of necroptosis modulation in renal diseases are still scarce. More clinical trials with larger sample sizes and longer follow-up periods are necessary to assess the safety, efficacy, and long-term outcomes of targeting necroptosis in the clinical setting.

Translational Potential and Clinical Implications of Targeting Necroptosis in Renal Diseases

Personalized medicine: Understanding the specific molecular mechanisms and cell type-specific regulators of necroptosis in renal diseases can pave the way for personalized treatment strategies. By identifying biomarkers or genetic profiles associated with necroptosis susceptibility, clinicians can tailor therapies to individual patients, optimizing treatment outcomes.

Combination therapies: Combining necroptosis-targeted therapies with existing treatment modalities, such as anti-inflammatory agents or immunosuppressive drugs, may offer synergistic effects and improved therapeutic outcomes. Integrating necroptosis modulation into existing treatment strategies could enhance their efficacy and reduce the burden of renal diseases.

Early intervention and prevention: Targeting necroptosis at early stages of renal diseases, such as acute kidney injury or early stages of chronic kidney disease, may prevent or delay disease progression. Early identification of patients at risk of necroptosis-mediated renal damage, and implementing targeted interventions could potentially halt or slow the development of irreversible renal injury.

## Conclusions

In conclusion, necroptosis is emerging as a regulated form of cell death with significant implications for renal health and disease. Through this review, we have provided an overview of necroptosis, highlighting its molecular signaling pathways and key players, and discussed its role in renal physiology and various renal diseases. Necroptosis, as a physiological process, plays a crucial role in normal renal development and tissue homeostasis. However, dysregulated necroptosis can contribute to tissue damage, inflammation, and fibrosis in renal diseases such as acute kidney injury, chronic kidney disease, and renal transplant rejection. Understanding the intricate interplay between necroptosis and inflammation in the kidney has shed light on potential therapeutic targets for intervention. The development of strategies to modulate necroptosis represents a promising avenue for the treatment of renal diseases. Inhibiting key mediators of necroptosis, targeting upstream regulators, and modulating the inflammatory response associated with necroptosis are potential therapeutic approaches. Small-molecule inhibitors and genetic interventions aimed at blocking necroptotic pathways have shown promise in preclinical models, but further clinical studies are needed to assess their efficacy and safety in humans. Continued research in the field of necroptosis and renal health is essential. Further investigations are required to unravel the specific molecular mechanisms underlying necroptosis in different renal cell types, identify additional therapeutic targets, and overcome the limitations of current studies. Robust preclinical and clinical studies will validate the translational potential of targeting necroptosis and pave the way for personalized treatment approaches in renal diseases.
